# The Brain Fatigue Syndrome—Symptoms, Probable Definition, and Pathophysiological Mechanisms

**DOI:** 10.3390/jcm14103271

**Published:** 2025-05-08

**Authors:** Birgitta Johansson, Lars Rönnbäck

**Affiliations:** Institute of Neuroscience and Physiology, Department of Clinical Neuroscience, Sahlgrenska Academy, University of Gothenburg, 40530 Göteborg, Sweden; lars.ronnback@neuro.gu.se

**Keywords:** brain, fatigue, syndrome, brain injury, neurological disorder, inflammatory disease, inflammatory reactive astrocytes, glutamate signaling, glucose uptake

## Abstract

Fatigue is a common consequence of traumatic brain injury, neurological diseases or developmental disorders, and systemic inflammatory diseases, including autoimmune conditions that affect the brain. This condition is characterized by reduced endurance for cognitive tasks, diminished quality of life, and impaired work capacity. In addition to cognitive difficulties, individuals often experience disproportionately long recovery times after demanding tasks, emotional instability, stress sensitivity, sensory sensitivity, impaired ability to initiate activities, and sleep disturbances. Tension headaches frequently occur when the brain is excessively activated by mental activity. In this paper, we propose the term “Brain Fatigue Syndrome” (BFS) as a collective name for the symptoms closely associated with this pathological fatigue resulting from brain impact. BFS can be identified through interviews and measured using the self-assessment instrument, the Mental Fatigue Scale (MFS). We suggest potential underlying mechanisms at the cellular level for the BFS symptom complex, including astrocyte dysfunction with impaired glutamate signaling and glucose uptake, mitochondrial dysfunction, blood–brain barrier dysfunction, and the activation of microglia and mast cells. In conclusion, BFS suggests a general brain impact. The symptoms associated with BFS typically resolve when the injury or disease heals. However, in some individuals, BFS persists even after the injury or illness has ostensibly healed.

## 1. Introduction

Brain dysfunction is common due to illness or injury affecting the Central Nervous System (CNS). It is well known that after an acquired brain injury or a brain disease, this dysfunction might give rise to cognitive, emotional, sensory, and sleeping problems [1,2,3]. The affected person experiences mental or brain fatigue [4]. The condition can cause difficulties for those affected when participating in everyday activities with a reduced quality of life [5,6,7,8] as well as a reduced ability to work [9,10,11]. There is no consensus on how to define this pathological mental or brain fatigue in humans, nor how to distinguish it from the normal fatigue that everyone can experience when the brain is drained of energy. Brain fog or cognitive fatigue is used by researchers with the intention to include cognitive difficulties in the concept of pathological fatigue [12], and central fatigue is used to stress the origin from the brain [13].

We propose Brain Fatigue Syndrome (BFS) as a collective name for the constellation of symptoms of brain dysfunction. It is a complex of symptoms consisting of cognitive, emotional, and sensory dysfunctions that are experienced by those affected by extreme fatigue mainly due to the increase in symptom intensity after mental activity. This is illustrated by a quote “The problem is that when I run out of energy, I am done for the day”.

We expand our previously proposed hypothesis on the underlying mechanisms at the cellular level where dysfunction in the astroglia support for glutamate transmission is in focus [14]. We also provide possible explanations for why the symptoms can be long-lasting. It is about inflammatory-reactive astrocytes using extracellular ATP signaling instead of intercellular Ca^2+^ signaling [15]. Extracellular ATP activates the purinergic receptors on microglial cells and astrocytes with the consequent production and release of inflammatory mediators [16]. The result is a vicious circle in which the glutamate uptake capacity of astrocytes is reduced.

## 2. Symptoms of BFS

Typical for BFS is a reduced ability to continue a mental activity repeatedly with a reduced ability to restore energy after mental activities. Those affected experience an unusually rapid loss of mental energy after mental activities. They need a long recovery time that is disproportionate to the level of effort. Furthermore, they report impaired attention and concentration over time, memory problems, slow thinking, problems initiating activities, emotional problems with tearfulness and/or irritability, sensitivity to stress, light, and noise, sleep problems, and usually headaches after overexertion. It is also common for sufferers to report a diurnal variation related to the activity performed. Symptoms are often less pronounced in the morning and worse later in the afternoon (Box 1 and Box 2).

Box 1 **What is Brain Fatigue Syndrome (BFS)?**

BFS is proposed as a state of disproportionately reduced mental energy that arises after mental activities and that has lasted at least one month, that is not cured with rest and sleep, and that has a score above 10 on the Mental Fatigue Scale (MFS). Usually, the recovery is disproportionately long, and it is common to have variations of the BFS symptoms over the day and between days.

Box 2 **Typical and associated symptoms of Brain Fatigue Syndrome (BFS).**

**Typical symptoms**

An unusual drain of mental energy after mental activity.

- Impaired attention and ability to concentrate over time.
- After overexertion, a long recovery time that is not proportional to the level of exertion.
- Daily variation in the fatigue symptoms where the fatigue is often better in the morning and worse in the afternoons and evenings. Often it varies from one day to the next.

**Associated symptoms**

- Problems with memory.
- Slowness of thinking.
- Reduced ability to start activities.
- Mood swings and tearfulness.
- Irritability.
- Sensitivity to stress.
- Sensitivity to light and noise.
- Sleep problems.
- Headache after overexertion.

## 3. Who May Suffer from BFS?

BFS may appear after a traumatic brain injury (TBI), a stroke including minor stroke, in multiple sclerosis (MS), Parkinson’s disease, brain tumor, after meningitis or encephalitis, but also in prolonged stress states, burnout syndrome, and myalgic encephalomyelopathy (ME) to name a few examples [17]. People with psychiatric diseases, major depression, or anxiety states may recognize this brain fatigue, as well as people suffering from developmental disorders [18]. Even people who suffer from rheumatic disease, psoriasis, or thyroid diseases often recognize this fatigue [19,20].

## 4. Self-Diagnostic Instrument for Brain Fatigue and BFS

**Figure 1:**
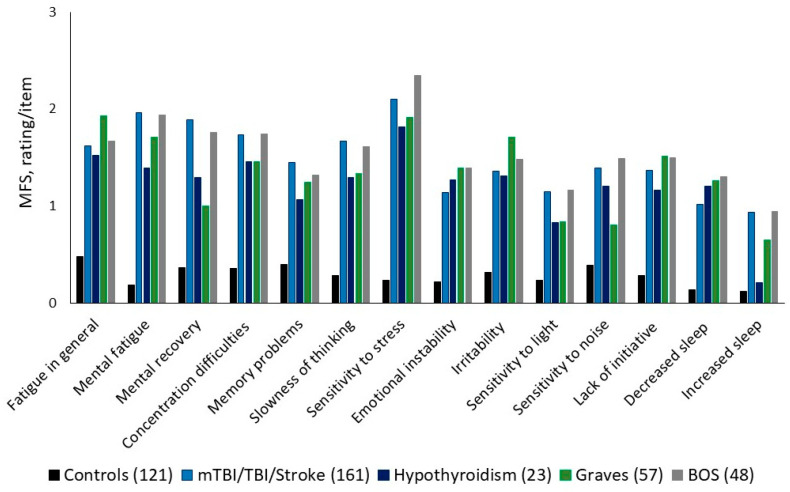
The figure shows how people suffering from BFS (Brain Fatigue Syndrome, MFS above cutoff) after an acquired brain injury (mild traumatic brain injury/mTBI/TBI/stroke); hypothyroidism; Graves’ disease; and burnout syndrome (BOS) compare to a control group’s response according to MFS (Mental Fatigue Scale).

**Figure 2:**
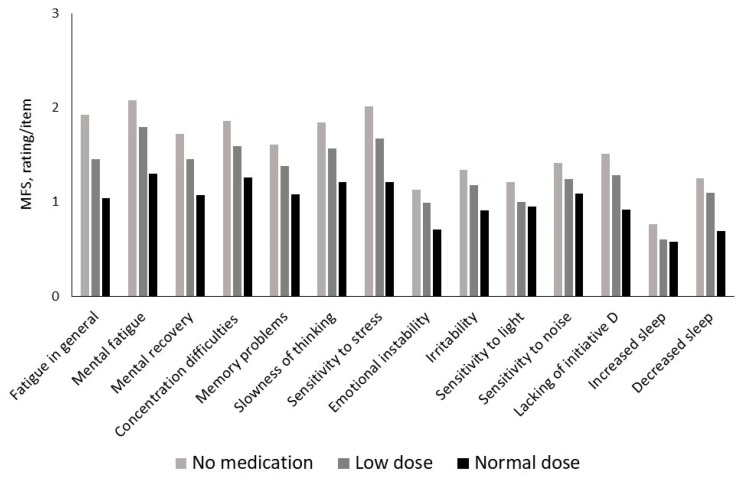
The figure shows how each single question from MFS is reported without treatment, with a low dose (15 mg/day) and a normal dose (60 mg/day) of methylphenidate. No drug shows the highest rating, low dose in-between, and normal dose with the lowest rating for all separate symptoms.

We developed the Mental Fatigue Scale (MFS), including all the symptoms mentioned above. The symptoms are closely connected within the frame of BFS, with a high internal correlation with Cronbach’s alpha of 0.944 and a total score cutoff above 10 [21,22]. This complex of symptoms is reported very similarly among different diagnostic groups who suffer from BFS. Figure 1 shows that BFS can be reported among a large group of people who had suffered, e.g., stroke, TBI, MS, endocrine diseases, and burnout syndrome and how they report their symptoms, when the total score of the MFS is above the cutoff (10.5 and above). For a comparison, a control group of healthy people is included in Figure 1 [9,20,21]. In addition, the symptoms vary in synchrony in response to pharmacological treatment, which is shown in Figure 2 from our study using methylphenidate for a group of people with BFS after a TBI. The figure shows the highest rating/more problems with no treatment, intermediate with a low dose (15 mg/day), and the lowest rating with the highest dose (60 mg/day) [23]. The MFS correlates with tests for processing speed work status, decreased social and leisure activities, and decreased brain activity in the frontal cortex and has been used for several diagnostic groups with very similar results [11,21,24,25,26].

## 5. Aspects of Different Symptoms Within BFS

### 5.1. Emotionality

Depression and anxiety correlate with brain fatigue [27], and can co-exist and also exist as separate states [20,28]. However, brain fatigue after a TBI is suggested to be related to the brain injury itself [7], rather than as a consequence of anxiety and depression [29], and did not improve with antidepressant treatment [30]. Fatigue also uniquely contributes to disability after a TBI, even after controlling for the injury severity, executive functions, and depression status [21,31]. Brain fatigue is also shown to be a separate state in the endocrine Graves’ disease and can exist without depression [20]. In summary, depression, anxiety, and brain fatigue correlate; some items overlap, but depression and brain fatigue should be regarded as separate states. In addition, emotional instability and irritability are significant symptoms related to BFS, and in the clinic, we have found that this can be alleviated with a low dose of antidepressant drugs, which, in fact, does not improve patients’ BFS.

### 5.2. Cognitive and Sensory Functions

The brain is continuously bombarded with information from the senses. Normally, there is a balance between focused attention and the suppression of competing information. The attention system can switch its focus back and forth as required and even manage multitasking. However, those suffering from BFS report problems managing the overwhelming inflow of information, and they lack the ability to supress irrelevant information. The consequence is reduced concentration, being easily distracted, one’s attention is easily lost, and patients cannot follow and adequately remember conversations. They cannot filter out background noise, such as a fan, or other disturbing noise and visual information. Normally, the sensory gating system or the filter system in the brain inhibits redundant or not relevant information from the environment, so a fan will not be a disturbing noise anymore [32,33]. Those suffering from BFS also have problems with automatic activities, such as reading for meaning. In addition, they complain of impaired memory, but from clinical experience, most often memory tests demonstrate minimal or no reduction in memory. Complex and basic processing speed is important in all higher cognitive functions, such as learning, memory, attention, problem solving, visuospatial function, and executive function and is the most sensitive cognitive construct to be impaired after a brain injury [34]. A reduced processing speed at the group level is reported to be related to fatigue [24,35,36].

### 5.3. Neurotransmitters in Relation to BFS

Glutamate is the most important excitatory neurotransmitter in the CNS and has a vital role in learning, memory, and mood regulation [37,38]. Glutamate in the prefrontal cortex has also been suggested to be a brain marker of brain fatigue [39]. Gamma-aminobutyric acid (GABA), probably the most important inhibitory transmitter, has been shown to be involved in cognitive impairments [40,41] and may be important for information suppression [42]. Dopamine selectively inhibits (GABA neurons) or enhances (glutamate neurons) synaptic signaling that supports action, attention, motivation and affect, and balanced neural activity [43]. Dopamine is essential for working memory, attention, and cognitive control [44]. Norepinephrine regulates a wide range of higher cognitive functions, including working memory, learning and attention, memory consolidation and retrieval, vigilance, and wakefulness. Furthermore, the dorsolateral prefrontal cortex (PFC) is sensitive to the levels of norepinephrine and dopamine, and norepinephrine is suggested to strengthen the PFC network connectivity, and dopamine decreases noise [45]. Acetylcholine is another neurotransmitter, important for attention, learning, and memory and to increase the signal relative to the noise [46]. Serotonin is a regulator of mood, sleep, and cognition [47]. After an experimental brain injury, a long-term downregulation of glutamate, GABA, acetylcholine, norepinephrine, dopamine, and serotonin was reported [48]. Brain fatigue was not specifically addressed in that report, but brain injuries commonly result in lasting BFS in humans, and the neurotransmitters are involved in cognitive, sensory, and emotional functions. Here, we discuss the connection between neurobiology and BFS and suggest a model for this.

## 6. Probable Pathophysiology of BFS at the Cellular Level

**Figure 3:**
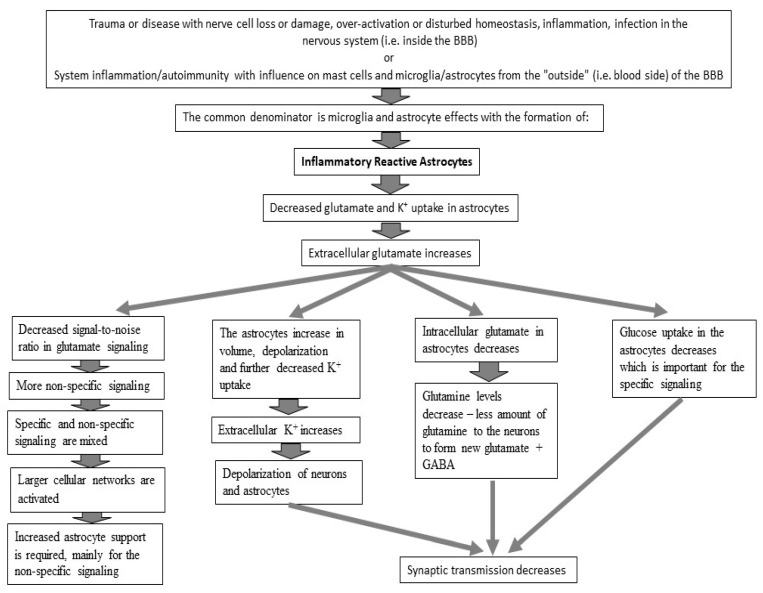
Schematic figure of probable cellular events underlying BFS. Trauma or disease in the nervous system causes neuroinflammation. The astrocytes become inflammatory reactive, whereby glutamate uptake and K^+^ buffering are reduced. Extracellular glutamate and K^+^ levels increase slightly. The astrocytes increase slightly in volume, and the cells’ membrane potential decreases due to the increased extracellular K^+^ levels. The membrane potential of neurons is also reduced, which might lead to reduced synaptic transmission. More non-specific signaling due to slightly increased extracellular glutamate leads to activation of larger cell networks, some of which are non-specific. Reduced glutamate uptake in the astrocytes leads to a reduction in the amount of glutamate in the astrocytes, which results in less glutamine being formed, and glucose transport to the astrocytes may be reduced.

We have previously suggested that brain fatigue may be a consequence of impaired glutamate signaling in the brain, since cognitive dysfunction is central in this pathological fatigue, and glutamate transmission is of crucial importance for cognitive processes such as information processing and the storage of information in the memory [14,49]. Here, we link the biochemical mechanisms to the symptoms included in BFS, illustrated in Figure 3 and in the text below.

### 6.1. Glutamate Transmission

Glutamate is the most abundant excitatory neurotransmitter in the brain [37]. It is stored in the synaptic vesicles of nerve terminals until it is released, and after the interaction with glutamate receptors on the postsynaptic membrane, excess glutamate is cleared from the synaptic cleft by astroglial cells to enable new and novel high-precision glutamate signaling with a high signal-to-noise ratio [50]. In the astrocytes, glutamate can then be metabolized via the tricarboxylic acid cycle and be used in protein synthesis or be converted to glutamine and transferred back to the neuron [51]. Astrocytes, together with neurons, are thus the key cells in glutamate transmission. The astrocytes form large cell networks. They have long processes where some enclose the glutamate synapses and others have contact with the walls of the small blood vessels and, together with endothelial cells, build up part of the blood–brain barrier (BBB) [52]. In addition to regulating the homeostasis of ions and other substances in the extracellular environment, astrocytes are responsible for the energy supply to the nerve cells and synapses [53].

### 6.2. Energy to the Brain

Glucose is the main source of energy, and a constant supply is needed for normal brain function [54]. Synaptic transmission is very energy intensive [55], and the brain is sensitive to disturbances of the glucose supply [56]. The glucose metabolism in the brain can be studied using positron emission tomography [(18)F]fluorodeoxyglucose (FDG-PET). From a review, sustained brain hypometabolism or reduced brain uptake using FDG-PET days to months after a TBI was demonstrated [57]. However, fatigue was not discussed in that review, but is a common long-term problem after a TBI. Furthermore, with use of FDG-PET, the fatigue in Parkinson’s disease, which is also a commonly reported fatigue [58], was linked to the depressed metabolism in the brain areas important for cognition and emotion [59], and the fatigue in Parkinson’s disease was suggested to be related to metabolic abnormalities and impaired functional interactions between the brain regions [60]. Glucose metabolism was significantly lower in MS and was suggested to be linked to fatigue [61]. With brain imaging methods (fMRI and fNIRS), reduced or altered neuronal activation in response to cognitive activity has also been demonstrated [26,62,63,64,65].

Astrocytes gain energy mostly via glycolysis, while for neurons, the energy supply is primarily via mitochondrial oxidative metabolism. The energy utilization in neurons is related to the activity of ion pumps for establishing electrical gradients, which is important for efficient neuronal activation and information transfer. Astrocytes are thus the key cells for the coupling between synaptic activity and energy metabolism transfer of lactate to neurons [53]. Both astrocytes and neurons are metabolically upregulated in response to increased neurotransmission [55]. This is at least to some extent regulated via glutamate signaling. For fast and specific synaptic transmission, glutamate must be removed from the synaptic cleft. For this process, glutamate is taken up in astrocytes. These cells clear the synaptic region from glutamate when the synaptic signaling is completed. The remaining glutamate level in the synapse region must be low for the continued signaling to occur with high precision (a high signal-to-noise ratio) [50] (Box 3).

Box 3 **Astrocyte networks.**

Actively involved in glutamate transmission.

Regulate the homeostasis of ions and substances in the extracellular milieu.

Provide nerve cells and synapses with an energy substrate.

(see also Siracusa et al., 2019) [66]

Once glutamate has been taken up into the astrocytes, there is an activation of glucose uptake from the blood to provide the astrocytes with glucose as energy building blocks, and this also occurs for neurons and synapses [53]. In addition to being an energy source, glucose is also a precursor for neurotransmitters, e.g., acetylcholine, glutamate, and GABA as well as neuromodulators [67]. The glutamate taken up by the astrocytes is converted into glutamine and transported back to the nerve cells for the synthesis of new glutamate in excitatory neurons or GABA in inhibitory neurons [51].

### 6.3. Reduced Glutamate Uptake

The impaired glutamate uptake into the astrocytes after synaptic transmission results in an increased extracellular concentration of glutamate, which can lead to excitotoxicity [68]. In the case of slightly increased extracellular glutamate levels, not reaching excitotoxic levels, glutamate may diffuse within the extracellular space and nearby neurons and give rise to the unspecific activation of such neurons. Thereby, larger cell networks could be activated in a non-specific way [69]. In addition, the astrocytes will increase in volume due to the slightly elevated extracellular glutamate concentration [70]. Astrocyte swelling leads to a reduced membrane potential with further swelling and reduced capacity for K^+^ buffering [71]. The situation at the cellular level might become untenable as specific and unspecific information-carrying signals are mixed.

## 7. BFS and Its Relation to Glutamate Signaling

A reduced amount of glutamate entering the astrocytes might reduce the glucose uptake from the blood and lower the energy availability in the synapses, at least for those involved in higher brain functions. This reduced glutamate level interacts with dopamine, noradrenalin, serotonin (5-HT), acetylcholine, and GABA, all of which are suggested to be reduced in the chronic phase after a brain injury [48], and involved in many diseases affecting the brain [72]. In support of this, emotional symptoms can be relieved by low-dose antidepressive drugs, and attention problems can be relieved by treatment with, e.g., methylphenidate, which increases dopamine and noradrenalin in the synaptic space in the frontal brain regions [23,73]. Reduced GABA might be important for information filtering in the brain [42], and a reduced GABA suppression of excitatory neural responses may lead to an overwhelming experience of impressions. BFS is exemplified below, with the related symptoms and neurotransmitters in a simplified manner, as intricate interactions between them occur.

- Cognitive difficulties such as concentration, memory, and learning relate to glutamate, acetylcholine, norepinephrine, and dopamine [37,38,43,44,46,74].
- Problems with motivation and initiating an activity might relate to dopamine [43].
- Emotional symptoms such as tearfulness and irritability might be related to serotonin and dopamine [47].
- Sensory sensitivity could be related to GABA and dopamine [42,45].

### Long-Lasting BFS

In some people, BFS remains for a long time after the injury or disease should have healed. A hypothetical explanation could be that astrocytes remain inflammatory reactive. It has been shown that inflammatory-activated astrocytes have disturbed intercellular Ca^2+^ signaling, and that they instead display extracellular ATP signaling [15]. ATP stimulates the purinergic receptors in microglia and astrocytes with the production of pro-inflammatory cytokines [16]. These cytokines can maintain neuroinflammation, which might remain self-maintaining and might cause the BFS symptoms to be long-lasting.

## 8. Discussion and Concluding Remarks

The healthy and well-functioning brain can manage cognitive activities for a relatively long time. However, everyone need breaks to recover and become alert and receptive again. When suffering from BFS, cognitive complaint is not the same as impaired cognitive function shown via neuropsychological tests [20,75]. BFS is about the endurance over time, the energy the person needs to accomplish cognitive tasks again and again, and managing all the impressions. When suffering from BFS, the ordinary activities are affected, and restoration takes a disproportionately long time. BFS can occur in diseases or damage to the nervous system, long-term stress such as burnout syndrome, in developmental disorders, but also in systemic inflammation and autoimmunity such as rheumatic disease, endocrinological diseases such as thyroid diseases, or skin diseases such as psoriasis. Normally, the BBB protects the brain from harmful substances, but when disrupted as in the case of brain injury or systemic inflammation, inflammatory mediators can cross the BBB [76,77] and activate microglia and astrocytes to become inflammatory reactive. Mast cell activation might also be of special interest [78].

Central to brain activity is high specificity and well-functioning energy utilization. Glucose is the primary source of energy, and as a simplified explanation, the astrocytes gain energy via glycolysis, and nerve cells and the synapses obtain energy via the mitochondria. Cognitive functions and emotions can be fully managed when the brain is intact with adequate homeostasis in different neuronal circuits. Thus, in the case of disturbed homeostasis or imbalance in the neural circuits, caused by injury or disease, the functions of the brain areas responsible for higher brain functions such as cognition and emotions are downregulated [55,79,80].

### 8.1. The Brain Fatigue Syndrome (BFS) Proposal

We suggest here BFS with symptoms that may seem unrelated, at least on superficial viewing. These are cognitive, emotional, and sensory symptoms. However, this symptom complex, included in BFS, seems robust based on clinical experience and could be diagnostic for brain impairment. The symptom complex can be present after injury or in diseases in the brain, or in the case of brain involvement caused by systemic inflammation and even in autoimmune diseases after the immune activation of brain cells across the BBB [77,81,82,83]. The symptoms included in BFS are very similar to the symptoms after a mild TBI/concussion (post-concussion syndrome). Fatigue is included in post-concussion syndrome, although fatigue is not given the prominence that we give it in BFS [2]. We emphasize the palpable mental fatigue as one of the main problems for those suffering from BFS, with reduced work ability and limitations in social and leisure activities, which have a major impact on the quality of life [5,6,7,8,10,11,24]. We show here that the symptoms within the symptom complex are closely linked (Figure 1). Brain fatigue has also been shown via brain-imaging methods to affect the brain function with reduced or altered activation in relation to fatigue [26,62,63,64,65]. BFS correlates with depression symptoms, but BFS and depression are different states [7,20,21,28], and for treatment and rehabilitation, both need to be dealt with, and adaptions are necessary [84].

We propose a reduced glutamate uptake capacity in astrocytes as a possible pathophysiological mechanism underlying BFS. Glutamate signaling is of central importance for cognitive processes. Glutamate signaling includes astrocytes’ fine regulation of extracellular glutamate, glutamate uptake with the associated signaling of the need for increased glucose uptake from the blood, and conversion of glutamate to glutamine for further transport to the nerve cells where the production of new glutamate from glutamine occurs in excitatory neurons and the production of GABA occurs in inhibitory neurons. Astrocytes are also responsible for the extracellular concentration (homeostasis) of ions, e.g., K^+^, but also other ions and substances.

A reduced glucose delivery from the blood to the astrocytes, which means reduced energy for specific synaptic transmission along with failing K^+^ buffering and the accompanying changes in the membrane potential of cells can lead to the reduced release of transmitters and thus reduced transmission in other transmitter systems as well.

The main source of energy is glucose, which is important for the synaptic transmission of cognitive functions and even other functions that originate in the brain. More research is needed for our proposed model with methods developed with higher precision and sensitivity for BFS.

### 8.2. Limitations

The proposed pathophysiology of BFS, involving brain astrocyte dysfunction, is hypothesized to be based on the results from extensive pre-clinical studies over the past 4–5 decades. This hypothesis can be further elucidated when high-resolution functional imaging techniques at the cellular level become available. We will then also obtain more information about the brain areas involved and whether BFS has a similar pathophysiology across different causes.

### 8.3. Conclusions

A limitation in energy availability, in combination with more energy-intensive synaptic transmission due to a mix of specific and non-specific information-carrying signals and thereby larger brain cell networks being activated, might lead to profound fatigue. In addition, there might be limitations or failure in the cognitive, sensory, and emotional systems. Together, these are the characteristics of the closely interconnected symptoms that are suggested to be part of BFS. By broadening and deepening the perspective on fatigue, including different patient groups, and moving from brain areas to the cellular level, our hypothesis and the research presented here can increase the knowledge about Brain Fatigue Syndrome and finally also the development of effective treatment options.

## Data Availability

Data/articles can be found at www.brainfatigue.se/publications and from articles refereed to in this article.
